# We need to aim at the top: Factors associated with cybersecurity awareness of cyber and information security decision-makers

**DOI:** 10.1371/journal.pone.0312266

**Published:** 2024-10-18

**Authors:** Simon Vrhovec, Blaž Markelj

**Affiliations:** Faculty of Criminal Justice and Security, University of Maribor, Ljubljana, Slovenia; Oakland University, UNITED STATES OF AMERICA

## Abstract

Cyberattacks pose a significant business risk to organizations. Although there is ample literature focusing on why people pose a major risk to organizational cybersecurity and how to deal with it, there is surprisingly little we know about cyber and information security decision-makers who are essentially the people in charge of setting up and maintaining organizational cybersecurity. In this paper, we study cybersecurity awareness of cyber and information security decision-makers, and investigate factors associated with it. We conducted an online survey among Slovenian cyber and information security decision-makers (*N* = 283) to (1) determine whether their cybersecurity awareness is associated with adoption of antimalware solutions in their organizations, and (2) explore which organizational factors and personal characteristics are associated with their cybersecurity awareness. Our findings indicate that awareness of well-known threats and solutions seems to be quite low for individuals in decision-making roles. They also provide insights into which threats (e.g., distributed denial-of-service (DDoS) attacks, botnets, industrial espionage, and phishing) and solutions (e.g., security operation center (SOC), advanced antimalware solutions with endpoint detection and response (EDR)/extended detection and response (XDR) capabilities, organizational critical infrastructure access control, centralized device management, multi-factor authentication, centralized management of software updates, and remote data deletion on lost or stolen devices) are cyber and information security decision-makers the least aware of. We uncovered that awareness of certain threats and solutions is positively associated with either adoption of advanced antimalware solutions with EDR/XDR capabilities or adoption of SOC. Additionally, we identified significant organizational factors (organizational role type) and personal characteristics (gender, age, experience with information security and experience with information technology (IT)) related to cybersecurity awareness of cyber and information security decision-makers. Organization size and formal education were not significant. These results offer insights that can be leveraged in targeted cybersecurity training tailored to the needs of groups of cyber and information security decision-makers based on these key factors.

## 1 Introduction

The multitude of cyberattacks and increasing cybercrime is increasingly leading organizations to accept cybersecurity as a significant business risk that can cause major financial loses, damage to reputation and legal liabilities [1–4]. The boundaries between information security and cyber security are essentially blurred in a highly connected society, such as the one we live in, and one cannot be practically achieved without the other. Information and cyber security decision-makers include information technology (IT)/information security (IS) executive (e.g., chief information security officer (CISO), chief information officer (CIO)), non-IT/IS executive (e.g., chief executive officer (CEO), chief financial officer (CFO)) and non-executive (e.g., IT administrator, department head) decision-makers. Decision-makers who are tasked with managing these cyber-risks need to deal with the challenges of evaluating risks in an evolving threat landscape and with constantly emerging new technologies [5, 6]. It is already challenging how to determine the real causes of cyber-incidents [7] which makes identifying the right countermeasures before incidents happen, taking into account their cost-benefit relationships, an exceedingly difficult task [8, 9]. Although a systematic approach to cybersecurity seems imperial, there are ideas about less measuring cybersecurity and more communicating about it [10]. For example, organizational leaders, such as CIO, CISO, and chief technology officer (CTO), are at the core of supporting cybersecurity strategies by improving governance and integration as well as fostering a new cultural mindset for cyber-resiliency [11]. Nevertheless, decision-makers are primarily tasked with decisions on cybersecurity measures that can range from adopting cybersecurity standards, technical measures, such as advanced antimalware [12] and intrusion detection [13] solutions, and human-centric measures, such as various types of cybersecurity training [14, 15], to inter-organizational measures, such as cyberthreat intelligence [16–19].

The human aspect of cybersecurity is a thriving research area. Studies focus on social engineering and phishing [20, 21], decision-making processes of security operation center (SOC) analysts [22], security concerns associated with adoption of social robots [23], effects of cyberattack proximity [24], fake and real news decision-making [25], information seeking [26], testing cyber soldiers [27], replacing aging and thus insecure smart devices [28], etc. These are just a few examples which paint the diversity of these studies. The published literature however seldom focuses on decision-makers even though they are among the key enablers of cybersecurity in organizations. For example, Bongiovanni et al. (2022) [29] investigated how decision-makers implement measures recommended by published cybersecurity guidelines. A study on adoption of cybersecurity standards in small and medium-sized enterprises (SMEs) found significant factors, such as demographics (organization size, intensity of IT usage, number of IT staff, number of IT security staff, investments in IT, investments in IT security), attitudes towards organizational cybersecurity risks and customer cybersecurity needs [30]. Triplett (2022) [31] studied how cybersecurity is promoted by organizational leaders. Studies indicate that unintentional human factors which facilitate cyberattacks, include lack of support by leaders, lack of knowledge and skills, being not aware of severity and damage cyberattacks can have, complacency and naivety coupled with reluctance to learn or seek help, and cybersecurity fatigue [32]. Some studies indicate a lack of awareness and cybersecurity education by decision-makers [33, 34]. It is especially worrisome that decision-makers are often unaware of what solutions can do to protect data [34]. Formal education and organization size were found to be contributing factors [34]. Nevertheless, we found a single study that suggests cyberthreat awareness among decision-makers is high [35] albeit it remains unclear how the researchers came to this conclusion since they did not report it in their work. The findings found in the literature are thus mixed at best. The literature also does not provide any insights into which factors may be associated with cybersecurity awareness of decision-makers. These insights may would to improve the awareness of cyber and information security decision-makers, e.g., with targeted cybersecurity training tailored to the needs of groups of decision-makers based on these key factors.

In this paper, we focus on cybersecurity awareness of decision-makers by investigating their awareness of well-known threats and solutions. First, we investigate whether there are differences between cybersecurity awareness of decision-makers in organizations adopting advanced antimalware solutions, and in organizations that do not. We break down advanced antimalware solutions into (1) advanced antimalware solutions with endpoint detection and response (EDR)/extended detection and response (XDR) capabilities, and (2) SOC as two of the most common advanced antimalware solutions found on the market today. This enabled us to determine whether cybersecurity awareness might play an important role in their decisions on cybersecurity. Second, we explored which organizational factors and personal characteristics are associated with cybersecurity awareness of decision-makers. Based on this, we developed these research questions:

*RQ1*: Are there differences in cybersecurity awareness of decision-makers across groups based on adoption of antimalware solutions in their organizations?
*RQ2a*: Are there differences in cybersecurity awareness of decision-makers across groups based on organizational factors?
*RQ2b*: Are there differences in cybersecurity awareness of decision-makers across groups based on their personal characteristics?

This paper is structured as follows. We present the research methodology in Section 2. The results of our study are presented in Section 3. We provide theoretical and practical implications as well as limitations and directions for future work in Section 4.

## 2 Methodology

### 2.1 Research design

We employed a cross-sectional research design to capture cybersecurity awareness of decision-makers and other variables at a specific time period. We conducted a survey among cyber and information security decision-makers in Slovenian organizations through the Cint platform (https://www.cint.com/). We included IT/IS executive (e.g., CISO, CIO), non-IT/IS executive (e.g., CEO, CFO) and non-executive (e.g., IT administrator, department head) decision-makers in the sample to cover the whole spectrum of cyber and information security decision-makers in organizations of different sizes, and enable comparison between them.

### 2.2 Ethical considerations

This study involved human participants. The study proposal was approved on 27 February 2023 by the Research Ethics Committee of the Faculty of Criminal Justice and Security at the University of Maribor [2702–2023]. We obtained written informed consent from the participants.

### 2.3 Measurement instrument

The survey questionnaire was designed to measure *awareness of threats*, *awareness of solutions*, *adopted antimalware solution type* and *adopted SOC realization* in addition to organizational factors (*organizational role type* and *organization size*) and personal characteristics (*gender*, *formal education*, *age*, *experience with information security* and *experience with IT*).

We developed a set of common cyber threats and a set of solutions for managing cyber-risks based on existing literature. We would like to note that both cyber threats and solutions are constantly evolving. Additionally, varying often conflicting taxonomies [36] make it exceedingly hard to capture all threats and solutions in manageable sets [37]. We thus opted to develop sets of threats and solutions that were common at the time the study was done [38–40]. *Awareness of threats* included questions on nine common threats: loss of access to data (e.g., ransomware, locking of devices) [41–44]; information system intrusion (e.g., hacking) [42, 44, 45]; theft of business-critical data (industrial espionage) [36, 45]; distributed denial of service (DDoS) attacks [41, 42, 45, 46]; takeover of devices (e.g., botnets) [42, 46]; malware infection (e.g., viruses, worms, Trojans, spyware) [42, 45]; phishing [41, 42, 45]; online fraud (e.g., CEO fraud, business email compromise) [42]; and internal threats (e.g., deliberate data deletion, unauthorized data access, unauthorized personal devices) [36, 41–45]. *Awareness of solutions* included questions on 12 available solutions for managing cyber-risks: remote data deletion on lost or stolen devices [43]; advanced antimalware solutions (with EDR/XDR capabilities) [42, 45]; secure connection (e.g., virtual private network (VPN)) [42, 45]; cloud synchronization of data [42, 43]; data backup [43, 45]; centralized device management, including mobile device management (MDM) [43]; advanced firewalls (with IPS/IDS capabilities) [41, 42, 45]; training on secure use of devices [42, 43]; multi-factor authentication (e.g., two-factor authentication (2FA)) [42]; security operation center (SOC) 24/7 [42]; centralized management of software updates [41, 45]; and organizational critical infrastructure access control [41].

*Awareness of threats* and *awareness of solutions* were measured on 4-point ordinal scales adapted from [47]: 1—“I am very familiar with this [threat/solution] and what it is”; 2—“I have heard about this [threat/solution] previously and am somewhat familiar with it”; 3—“I have heard mention of this [threat/solution] before but am largely unfamiliar with it”; 4—“I was not aware of this [threat/solution] before today”.

Respondents were first shown a description of advanced antimalware solutions, including descriptions of EDR and XDR capabilities as well as SOC. Then, respondents were asked which types of antimalware solutions and SOC realizations were adopted in their respective organizations. Options for *adopted antimalware solution type* included an advanced antimalware solution with EDR/XDR capabilities, a standard antimalware solution, such as antivirus programs, and none. Options for *adopted SOC realization* included a dedicated 24/7 security team within a respondent’s organization, an external SOC (SOC-as-a-service model), and none (incidents handled by IT or information security department).

### 2.4 Sample and data collection

The survey was conducted from 13 to 20 March 2023 through the Cint platform. We used organizational role as a screening question to ensure that all respondents were qualified to take the survey. A total of 356 respondents took the survey. After excluding unqualified respondents, such as decision-makers with no role in information and cyber security-related decisions (21), responses with over 10 percent missing values (5), and responses that indicated non-engagement bias (47), we were left with *N* = 283 useful responses. Table 1 presents the key sample characteristics.

**Table 1:** Sample characteristics.

Characteristic		Frequency	Percent
Organization size	Micro	133	47.0%
	Small	88	31.1%
	Medium	49	17.3%
	Large	13	4.6%
Role	CEO	78	27.6%
	CFO	36	12.7%
	CIO	50	17.7%
	IT executive	33	11.7%
	CISO	22	7.8%
	Other	64	22.6%
Gender	Female	87	30.7%
	Male	195	68.9%
	N/A	1	0.4%
Formal education	Finished high school	54	19.1%
	Bachelor’s degree	90	31.8%
	Master’s degree	106	37.5%
	PhD degree	33	11.7%

Average respondent *age* was 36.7 years (*SD* = 12.1). Mean *experience with IT* was 10.4 years (*SD* = 8.5), and mean *experience with information security* was 9.9 years (*SD* = 8.1). Average *employment duration* was 10.4 years (*SD* = 9.1).

Decision-makers represented organization from various industries. Table 2 presents which industries were represented in the study.

**Table 2:** Organization industries.

Industry	Frequency	Percent
Information and communication	36	12.7%
Wholesale and retail trade; Repair of motor vehicles and motorcycles	28	9.9%
Construction	23	8.1%
Professional, scientific and technical activities	20	7.1%
Other service activities	20	7.1%
Electricity, gas, steam and air conditioning supply	18	6.4%
Water supply; Sewerage, waste management and remediation activities	16	5.7%
Transportation and storage	14	4.9%
Manufacturing	13	4.6%
Financial and insurance activities	13	4.6%
Agriculture, forestry and fishing	11	3.9%
Accommodation and food service activities	11	3.9%
Administrative and support service activities	11	3.9%
Education	11	3.9%
Arts, entertainment and recreation	9	3.2%
Human health and social work activities	8	2.8%
Real estate activities	7	2.5%
Mining and quarrying	5	1.8%
Public administration and defence; Compulsory social security	3	1.1%
N/A	6	2.1%

### 2.5 Data analysis

We used R version 4.4.1 with packages *psych* version 2.4.6.26, *dplyr* version 1.1.4, *FSA* version 0.9.5, *car* version 3.1–2 and *ltm* version 1.2–0 for data analysis. We merged some categories and scores before data analysis. First, we merged categories for *organizational role* into categories IT/IS executive (e.g., CISO, CIO), non-IT/IS executive (e.g., CEO, CFO) and non-executive (e.g., IT administrator, department head). This enabled us to test whether the *organizational role type* according to IT/IS background and/or position within an organization is associated with cybersecurity awareness of decision-makers. Next, we tested the overall reliability of *awareness of threats* and *awareness of solutions* as theoretical constructs with Cronbach’s alpha (CA). CA was 0.876 and 0.884 for *awareness of threats* and *awareness of solutions*, respectively, indicating good reliability. Finally, we merged scores for *awareness of threats* and *awareness of solutions* to improve the reliability of measuring individual dimensions of these constructs. Measuring awareness with self-reported questionnaires is challenging as people do not tend to be very realistic about their own awareness of specific phenomena. We used a scale that adequately addresses this issue by describing four clearly distinct levels of awareness. We decided to further improve the reliability of the used scale by dividing respondents into those that are at least somewhat familiar with a threat/solution (*aware*) and those who are largely unfamiliar with it or heard about it for the first time during the survey (*not aware*). A beneficial side-effect of this mergence is an improved interpretability of the study results enabling the comparison of the awareness level of respondents in different groups.

Data analysis included non-parametric tests, such as Kruskal-Wallis test for determining differences between three or more groups with post-hoc Dunn’s tests with Bonferroni correction for determining differences between pairs of those groups. We used Wilcoxon tests to determine differences between two groups. We also used parametric tests when possible—we conducted independent samples *t* tests for determining differences in means across two groups. We used standard significance levels (*α* = 0.05, *α* = 0.01, and *α* = 0.001) for determining significance of all statistical test results.

## 3 Results

In this section, we present the results relevant for answering the posed research questions. We analyzed the data related to cybersecurity awareness of decision-makers separately for all research questions.

### 3.1 RQ1: Are there differences in cybersecurity awareness of decision-makers across groups based on adoption of antimalware solutions in their organizations?

We analyzed cybersecurity awareness of cyber and information security decision-makers separately for two of the most common advanced antimalware solutions found on the market today: (1) advanced antimalware solutions with EDR/XDR capabilities, and (2) security operation centers (SOC). Since the former is a technical solution, we compared it with other types of technical solutions (i.e., a standard antimalware solution and none). The latter is an organizational solution which can have varying realizations (i.e., internal SOC, external SOC, none).

#### 3.1.1 Adopted antimalware solution type

Table 3 presents the differences in respondents’ *awareness of threats* for groups based on their organization’s *adopted antimalware solution type*. The results indicate significant differences for awareness of six threats. However, there were only four threats with clearly distinguishable differences between different types of adopted antimalware solutions. For three of these threats (i.e., industrial espionage, botnets and phishing), there were significant differences between organizations that have either antimalware solution (EDR/XDR or standard) and those which do not. In all cases, respondents in the latter had a significantly lower awareness of these threats. Awareness of DDoS attacks was however significantly higher for respondents in organizations adopting advanced antimalware solutions with EDR/XDR capabilities than respondents in those adopting a standard antimalware solution or not adopting any at all.

**Table 3:** Differences in awareness of threats across adopted antimalware solution types.

	1: EDR/XDR	2: Standard	3: None	*H*	*p*	*p*_1−2_	*p*_1−3_	*p*_2−3_
Loss of access to data (e.g., ransomware, locking of devices)								
Not aware	10(13.0%)	26(19.0%)	14(33.3%)	7.032*	0.0297	0.9190	0.0250	0.1223
Aware	66(85.7%)	111(81.0%)	28(66.7%)					
Information system intrusion (e.g., hacking)								
Not aware	14(18.2%)	18(13.1%)	8(19.0%)	1.392	0.4986			
Aware	63(81.8%)	119(86.9%)	34(81.0%)					
Theft of business-critical data (industrial espionage)								
Not aware	18(23.4%)	34(24.8%)	20(47.6%)	9.459**	0.0088	1.0000	0.0151	0.0123
Aware	59(76.6%)	103(75.2%)	22(52.4%)					
Distributed denial of service (DDoS) attacks								
Not aware	11(14.3%)	56(40.9%)	21(50.0%)	20.335***	0.0000	0.0003	0.0003	0.8324
Aware	65(84.4%)	81(59.1%)	21(50.0%)					
Takeover of devices (e.g., botnets)								
Not aware	15(19.5%)	41(29.9%)	22(52.4%)	13.537**	0.0011	0.3474	0.0007	0.0193
Aware	61(79.2%)	95(69.3%)	20(47.6%)					
Malware infection (e.g., viruses, worms, Trojans, spyware)								
Not aware	9(11.7%)	19(13.9%)	6(14.3%)	0.180	0.9137			
Aware	66(85.7%)	118(86.1%)	36(85.7%)					
Phishing								
Not aware	11(14.3%)	31(22.6%)	18(42.9%)	12.422**	0.0020	0.5028	0.0013	0.0207
Aware	66(85.7%)	106(77.4%)	24(57.1%)					
Online fraud (e.g., CEO fraud, business email compromise)								
Not aware	15(19.5%)	30(21.9%)	10(23.8%)	0.330	0.8477			
Aware	62(80.5%)	107(78.1%)	32(76.2%)					
Internal threats (e.g., deliberate data deletion, unauthorized data access, unauthorized personal devices)								
Not aware	8(10.4%)	30(21.9%)	14(33.3%)	9.263**	0.0097	0.1445	0.0087	0.2989
Aware	67(87.0%)	105(76.6%)	27(64.3%)					

We conducted Kruskal-Wallis tests to determine whether there are differences in awareness of threats across groups. For threats with significant differences across groups, we conducted post-hoc Dunn’s tests with Bonferroni correction to determine which groups significantly differ from each other.

Notes:

*p < 0.05,

**p < 0.01,

***p < 0.001.

Table 4 presents the differences in respondents’ *awareness of solutions* for groups based on their organization’s *adopted antimalware solution type*. The results indicate significant differences for awareness of 10 solutions. There were however just eight clear differences between different types of adopted antimalware solutions. For six of these solutions (i.e., remote deletion, advanced firewalls, training, multi-factor authentication, SOC and centralized management of software updates), there were significant differences between respondents in organizations that have either antimalware solution (EDR/XDR or standard) and respondents in those which do not. In all cases, respondents in the latter had significantly lower awareness of these solutions. For two solutions (i.e., advanced antimalware solutions with EDR/XDR capabilities and centralized device management), there were clear differences among all three adopted antimalware solution type groups. In both cases, the most aware were respondents in organizations adopting advanced antimalware solutions with EDR/XDR capabilities, followed by respondents in those which adopted a standard antimalware solution, while the least aware were respondents in organizations that did not adopt any antimalware solution.

**Table 4:** Differences in awareness of solutions across adopted antimalware solution types.

	1: EDR/XDR	2: Standard	3: None	*H*	*p*	*p*_1−2_	*p*_1−3_	*p*_2−3_
Remote data deletion on lost or stolen devices								
Not aware	12(15.6%)	34(24.8%)	19(45.2%)	12.081**	0.0024	0.4587	0.0016	0.0265
Aware	63(81.8%)	102(74.5%)	23(54.8%)					
Advanced antimalware solutions (with EDR/XDR capabilities)								
Not aware	9(11.7%)	53(38.7%)	27(64.3%)	35.845***	0.0000	0.0002	0.0000	0.0055
Aware	67(87.0%)	82(59.9%)	14(33.3%)					
Secure connection (e.g., VPN)								
Not aware	15(19.5%)	23(16.8%)	6(14.3%)	0.546	0.7611			
Aware	62(80.5%)	114(83.2%)	36(85.7%)					
Cloud synchronization of data								
Not aware	15(19.5%)	16(11.7%)	13(31.0%)	8.624*	0.0134	0.4590	0.3426	0.0123
Aware	62(80.5%)	120(87.6%)	29(69.0%)					
Data backup								
Not aware	13(16.9%)	20(14.6%)	9(21.4%)	1.072	0.5850			
Aware	63(81.8%)	116(84.7%)	33(78.6%)					
Centralized device management, including mobile device management (MDM)								
Not aware	7(9.1%)	38(27.7%)	22(52.4%)	26.585***	0.0000	0.0075	0.0000	0.0057
Aware	70(90.9%)	97(70.8%)	20(47.6%)					
Advanced firewalls (with IPS/IDS capabilities)								
Not aware	12(15.6%)	24(17.5%)	18(42.9%)	14.229***	0.0008	1.0000	0.0015	0.0015
Aware	65(84.4%)	112(81.8%)	24(57.1%)					
Training on secure use of devices								
Not aware	9(11.7%)	28(20.4%)	16(38.1%)	11.260**	0.0036	0.4009	0.0024	0.0446
Aware	67(87.0%)	108(78.8%)	26(61.9%)					
Multi-factor authentication (e.g., 2FA)								
Not aware	12(15.6%)	30(21.9%)	27(64.3%)	36.207***	0.0000	0.9234	0.0000	0.0000
Aware	65(84.4%)	106(77.4%)	15(35.7%)					
Security operation center (SOC) 24/7								
Not aware	16(20.8%)	52(38.0%)	28(66.7%)	23.445***	0.0000	0.0517	0.0000	0.0024
Aware	59(76.6%)	85(62.0%)	14(33.3%)					
Centralized management of software updates								
Not aware	11(14.3%)	35(25.5%)	22(52.4%)	21.232***	0.0000	0.2111	0.0000	0.0012
Aware	66(85.7%)	101(73.7%)	19(45.2%)					
Organizational critical infrastructure access control								
Not aware	13(16.9%)	38(27.7%)	19(45.2%)	10.445**	0.0054	0.2997	0.0037	0.0865
Aware	62(80.5%)	98(71.5%)	23(54.8%)					

We conducted Kruskal-Wallis tests to determine whether there are differences in awareness of solutions across groups. For solutions with significant differences across groups, we conducted post-hoc Dunn’s tests with Bonferroni correction to determine which groups significantly differ from each other.

Notes:

*p < 0.05,

**p < 0.01,

***p < 0.001.

These results indicate that awareness of certain threats and solutions is positively associated with adoption of antimalware solutions. Decision-makers in organizations adopting an advanced antimalware solution with EDR/XDR capabilities are more aware of one threat (i.e., DDoS attacks) and two solutions (i.e., advanced antimalware solutions with EDR/XDR capabilities and centralized device management) than decision-makers in organizations adopting a standard antimalware solution. Additionally, they are more aware of four threats (i.e., industrial espionage, botnets, phishing and DDoS attacks) and eight solutions (i.e., remote deletion, advanced firewalls, training, multi-factor authentication, SOC, centralized management of software updates, advanced antimalware solutions with EDR/XDR capabilities and centralized device management) than decision-makers in organizations which do not adopt any antimalware solution. Decision-makers in organizations adopting a standard antimalware solution are similarly more aware of three threats (i.e., industrial espionage, botnets and phishing) and eight solutions (i.e., remote deletion, advanced firewalls, training, multi-factor authentication, SOC, centralized management of software updates, advanced antimalware solutions with EDR/XDR capabilities and centralized device management) than decision-makers in organizations which do not adopt any antimalware solution.

#### 3.1.2 Adopted SOC realization

Table 5 presents the differences in respondents’ *awareness of threats* for groups based on *adopted SOC realization* in their organizations. The results indicate significant differences in awareness of five threats. A single threat (i.e., online fraud) had clearly distinguishable differences between different realizations of SOC. Respondents in organizations adopting an internal SOC were markedly more aware of this threat than respondents in organizations adopting an external SOC or not having a SOC.

**Table 5:** Differences in awareness of threats across adopted SOC realizations.

	1: Internal SOC	2: External SOC	3: None	*H*	*p*	*p*_1−2_	*p*_1−3_	*p*_2−3_
Loss of access to data (e.g., ransomware, locking of devices)								
Not aware	5(7.5%)	11(17.7%)	30(25.4%)	8.883*	0.0118	0.4237	0.0089	0.6301
Aware	61(91.0%)	51(82.3%)	88(74.6%)					
Information system intrusion (e.g., hacking)								
Not aware	7(10.4%)	11(17.7%)	18(15.3%)	1.454	0.4834			
Aware	60(89.6%)	51(82.3%)	100(84.7%)					
Theft of business-critical data (industrial espionage)								
Not aware	14(20.9%)	15(24.2%)	34(28.8%)	1.479	0.4773			
Aware	53(79.1%)	47(75.8%)	84(71.2%)					
Distributed denial of service (DDoS) attacks								
Not aware	11(16.4%)	21(33.9%)	48(40.7%)	11.144**	0.0038	0.1147	0.0026	1.0000
Aware	55(82.1%)	41(66.1%)	70(59.3%)					
Takeover of devices (e.g., botnets)								
Not aware	13(19.4%)	21(33.9%)	41(34.7%)	5.072	0.0792			
Aware	53(79.1%)	41(66.1%)	76(64.4%)					
Malware infection (e.g., viruses, worms, Trojans, spyware)								
Not aware	3(4.5%)	14(22.6%)	13(11.0%)	9.904**	0.0071	0.0057	0.5824	0.0787
Aware	63(94.0%)	48(77.4%)	104(88.1%)					
Phishing								
Not aware	7(10.4%)	20(32.3%)	27(22.9%)	9.067*	0.0107	0.0084	0.1491	0.4467
Aware	60(89.6%)	42(67.7%)	91(77.1%)					
Online fraud (e.g., CEO fraud, business email compromise)								
Not aware	3(4.5%)	15(24.2%)	33(28.0%)	14.964***	0.0006	0.0174	0.0005	1.0000
Aware	64(95.5%)	47(75.8%)	85(72.0%)					
Internal threats (e.g., deliberate data deletion, unauthorized data access, unauthorized personal devices)								
Not aware	6(9.0%)	10(16.1%)	27(22.9%)	5.759	0.0562			
Aware	59(88.1%)	52(83.9%)	89(75.4%)					

We conducted Kruskal-Wallis tests to determine whether there are differences in awareness of threats across groups. For threats with significant differences across groups, we conducted post-hoc Dunn’s tests with Bonferroni correction to determine which groups significantly differ from each other.

Notes:

*p < 0.05,

**p < 0.01,

***p < 0.001.

Table 6 presents the differences in respondents’ *awareness of solutions* for groups based on *adopted SOC realization* in their organizations. The results indicate significant differences in awareness of five solutions, with clear differences between different realizations of SOC for two of them. For both SOC and critical infrastructure access control, there were significant differences between respondents in organizations that have either realization of SOC (internal or external) and respondents in those which do not. The latter had significantly lower awareness of both solutions.

**Table 6:** Differences in awareness of solutions across adopted SOC realizations.

	1: Internal SOC	2: External SOC	3: None	*H*	*p*	*p*_1−2_	*p*_1−3_	*p*_2−3_
Remote data deletion on lost or stolen devices								
Not aware	7(10.4%)	13(21.0%)	34(28.8%)	8.144*	0.0171	0.5018	0.0134	0.6467
Aware	58(86.6%)	49(79.0%)	83(70.3%)					
Advanced antimalware solutions (with EDR/XDR capabilities)								
Not aware	12(17.9%)	23(37.1%)	45(38.1%)	8.899*	0.0117	0.0587	0.0136	1.0000
Aware	54(80.6%)	38(61.3%)	71(60.2%)					
Secure connection (e.g., VPN)								
Not aware	11(16.4%)	11(17.7%)	20(16.9%)	0.040	0.9800			
Aware	56(83.6%)	51(82.3%)	98(83.1%)					
Cloud synchronization of data								
Not aware	9(13.4%)	15(24.2%)	18(15.3%)	3.034	0.2194			
Aware	57(85.1%)	47(75.8%)	100(84.7%)					
Data backup								
Not aware	9(13.4%)	14(22.6%)	19(16.1%)	1.979	0.3717			
Aware	58(86.6%)	48(77.4%)	97(82.2%)					
Centralized device management, including mobile device management (MDM)								
Not aware	8(11.9%)	11(17.7%)	34(28.8%)	7.851*	0.0197	1.0000	0.0230	0.2423
Aware	58(86.6%)	51(82.3%)	83(70.3%)					
Advanced firewalls (with IPS/IDS capabilities)								
Not aware	10(14.9%)	10(16.1%)	27(22.9%)	2.103	0.3494			
Aware	56(83.6%)	52(83.9%)	91(77.1%)					
Training on secure use of devices								
Not aware	8(11.9%)	13(21.0%)	29(24.6%)	4.166	0.1246			
Aware	58(86.6%)	49(79.0%)	88(74.6%)					
Multi-factor authentication (e.g., 2FA)								
Not aware	11(16.4%)	19(30.6%)	32(27.1%)	3.740	0.1541			
Aware	55(82.1%)	43(69.4%)	86(72.9%)					
Security operation center (SOC) 24/7								
Not aware	12(17.9%)	18(29.0%)	57(48.3%)	18.626***	0.0000	0.6023	0.0001	0.0269
Aware	54(80.6%)	44(71.0%)	60(50.8%)					
Centralized management of software updates								
Not aware	11(16.4%)	14(22.6%)	33(28.0%)	3.443	0.1788			
Aware	56(83.6%)	48(77.4%)	83(70.3%)					
Organizational critical infrastructure access control								
Not aware	9(13.4%)	12(19.4%)	42(35.6%)	12.949**	0.0015	1.0000	0.0025	0.0437
Aware	57(85.1%)	50(80.6%)	74(62.7%)					

We conducted Kruskal-Wallis tests to determine whether there are differences in awareness of solutions across groups. For solutions with significant differences across groups, we conducted post-hoc Dunn’s tests with Bonferroni correction to determine which groups significantly differ from each other.

Notes:

*p < 0.05,

**p < 0.01,

***p < 0.001.

These results suggest that awareness of certain threats and solutions is positively associated with adoption of SOC albeit this association does not seem to be as diverse as its association with adoption of antimalware solutions. Decision-makers in organizations adopting an internal SOC are more aware of one threat (i.e., online fraud) than decision-makers in organizations adopting an external SOC. There are no differences in their awareness of any solutions. Also, they are more aware of one threat (i.e., online fraud) than decision-makers in organizations which do not have a SOC. Decision-makers in organizations adopting either realization of SOC (i.e., internal or external SOC) are more aware of two solutions (i.e., SOC and critical infrastructure access control) than decision-makers in organizations which do not have a SOC.

### 3.2 RQ2a: Are there differences in cybersecurity awareness of decision-makers across groups based on organizational factors?

#### 3.2.1 Organizational role type

Table 7 presents the differences in respondents’ *awareness of threats* for groups based on their *organizational role type*. The results indicate significant differences for awareness of six threats. However, a single threat (i.e., industrial espionage) had clearly distinguishable differences between different organizational role types. Non-IT/IS executive decision-makers were significantly less aware of this threat than IT/IS executive and non-executive decision-makers.

**Table 7:** Differences in awareness of threats across organizational role types.

	1: IT/IS executive	2: Non-IT/IS executive	3: Non-executive	*H*	*p*	*p*_1−2_	*p*_1−3_	*p*_2−3_
Loss of access to data (e.g., ransomware, locking of devices)								
Not aware	18(17.1%)	30(26.3%)	7(10.9%)	6.912*	0.0316	0.2413	0.9727	0.0358
Aware	87(82.9%)	83(72.8%)	57(89.1%)					
Information system intrusion (e.g., hacking)								
Not aware	15(14.3%)	24(21.1%)	2(3.1%)	10.602**	0.0050	0.4678	0.1378	0.0034
Aware	90(85.7%)	90(78.9%)	62(96.9%)					
Theft of business-critical data (industrial espionage)								
Not aware	22(21.0%)	46(40.4%)	9(14.1%)	17.539***	0.0002	0.0039	0.9894	0.0005
Aware	83(79.0%)	68(59.6%)	55(85.9%)					
Distributed denial of service (DDoS) attacks								
Not aware	26(24.8%)	50(43.9%)	23(35.9%)	9.064*	0.0108	0.0079	0.4216	0.7998
Aware	79(75.2%)	63(55.3%)	41(64.1%)					
Takeover of devices (e.g., botnets)								
Not aware	26(24.8%)	44(38.6%)	21(32.8%)	4.795	0.0910			
Aware	78(74.3%)	69(60.5%)	43(67.2%)					
Malware infection (e.g., viruses, worms, Trojans, spyware)								
Not aware	16(15.2%)	20(17.5%)	2(3.1%)	7.691*	0.0214	1.0000	0.0814	0.0210
Aware	89(84.8%)	93(81.6%)	61(95.3%)					
Phishing								
Not aware	25(23.8%)	37(32.5%)	7(10.9%)	10.288**	0.0058	0.4117	0.1775	0.0041
Aware	80(76.2%)	77(67.5%)	57(89.1%)					
Online fraud (e.g., CEO fraud, business email compromise)								
Not aware	25(23.8%)	26(22.8%)	8(12.5%)	3.514	0.1726			
Aware	80(76.2%)	88(77.2%)	56(87.5%)					
Internal threats (e.g., deliberate data deletion, unauthorized data access, unauthorized personal devices)								
Not aware	18(17.1%)	27(23.7%)	9(14.1%)	3.503	0.1735			
Aware	87(82.9%)	82(71.9%)	55(85.9%)					

We conducted Kruskal-Wallis tests to determine whether there are differences in awareness of threats across groups. For threats with significant differences across groups, we conducted post-hoc Dunn’s tests with Bonferroni correction to determine which groups significantly differ from each other.

Notes:

*p < 0.05,

**p < 0.01,

***p < 0.001.

Table 8 presents the differences in respondents’ *awareness of solutions* for groups based on their *organizational role type*. The results suggest significant differences for awareness of all 12 solutions. Nevertheless, only six had clear differences between different organizational role types. For four solutions (i.e., training, multi-factor authentication, centralized management of software updates and critical infrastructure access control), non-IT/IS executive decision-makers were significantly less aware of these solutions than IT/IS executive and non-executive decision-makers. Perhaps a bit surprisingly, non-executive decision-makers were significantly more aware of remote data deletion and cloud synchronization of data than both IT/IS and non-IT/IS executive decision-makers.

**Table 8:** Differences in awareness of solutions across organizational role types.

	1: IT/IS executive	2: Non-IT/IS executive	3: Non-executive	*H*	*p*	*p*_1−2_	*p*_1−3_	*p*_2−3_
Remote data deletion on lost or stolen devices								
Not aware	16(15.2%)	41(36.0%)	13(20.3%)	13.876***	0.0010	0.0010	1.0000	0.0496
Aware	88(83.8%)	71(62.3%)	51(79.7%)					
Advanced antimalware solutions (with EDR/XDR capabilities)								
Not aware	33(31.4%)	46(40.4%)	22(34.4%)	1.901	0.3866			
Aware	71(67.6%)	67(58.8%)	40(62.5%)					
Secure connection (e.g., VPN)								
Not aware	23(21.9%)	26(22.8%)	2(3.1%)	12.408**	0.0020	1.0000	0.0063	0.0032
Aware	82(78.1%)	88(77.2%)	62(96.9%)					
Cloud synchronization of data								
Not aware	21(20.0%)	25(21.9%)	3(4.7%)	9.365**	0.0093	1.0000	0.0304	0.0109
Aware	83(79.0%)	89(78.1%)	61(95.3%)					
Data backup								
Not aware	21(20.0%)	23(20.2%)	4(6.3%)	6.586*	0.0372	1.0000	0.0693	0.0545
Aware	84(80.0%)	90(78.9%)	59(92.2%)					
Centralized device management, including mobile device management (MDM)								
Not aware	19(18.1%)	37(32.5%)	19(29.7%)	6.157*	0.0460	0.0487	0.3145	1.0000
Aware	85(81.0%)	76(66.7%)	45(70.3%)					
Advanced firewalls (with IPS/IDS capabilities)								
Not aware	17(16.2%)	30(26.3%)	12(18.8%)	3.491	0.1746			
Aware	87(82.9%)	84(73.7%)	52(81.3%)					
Training on secure use of devices								
Not aware	22(21.0%)	31(27.2%)	7(10.9%)	6.231*	0.0444	0.8339	0.3764	0.0377
Aware	82(78.1%)	83(72.8%)	56(87.5%)					
Multi-factor authentication (e.g., 2FA)								
Not aware	21(20.0%)	40(35.1%)	14(21.9%)	7.101*	0.0287	0.0392	1.0000	0.1680
Aware	83(79.0%)	74(64.9%)	50(78.1%)					
Security operation center (SOC) 24/7								
Not aware	27(25.7%)	54(47.4%)	27(42.2%)	11.836**	0.0027	0.0025	0.0818	1.0000
Aware	78(74.3%)	59(51.8%)	36(56.3%)					
Centralized management of software updates								
Not aware	23(21.9%)	41(36.0%)	10(15.6%)	10.899**	0.0043	0.0425	1.0000	0.0072
Aware	82(78.1%)	71(62.3%)	54(84.4%)					
Organizational critical infrastructure access control								
Not aware	19(18.1%)	47(41.2%)	13(20.3%)	17.487***	0.0002	0.0003	1.0000	0.0080
Aware	86(81.9%)	65(57.0%)	50(78.1%)					

We conducted Kruskal-Wallis tests to determine whether there are differences in awareness of solutions across groups. For solutions with significant differences across groups, we conducted post-hoc Dunn’s tests with Bonferroni correction to determine which groups significantly differ from each other.

Notes:

*p < 0.05,

**p < 0.01,

***p < 0.001.

These results indicate some differences in decision-makers’ awareness of certain threats and solutions depending on their organizational role type. Non-IT/IS executive decision-makers are less aware of one threat (i.e., industrial espionage) and four solutions (i.e., training, multi-factor authentication, centralized management of software updates and critical infrastructure access control) than both IT/IS executive and non-executive decision-makers. They are also less aware of two solutions (i.e., remote data deletion and cloud synchronization of data) than non-executive decision-makers. IT/IS executive decision-makers are additionally less aware of these two solutions than non-executive decision-makers.

#### 3.2.2 Organization size

Table 9 shows the differences in respondents’ *awareness of threats* for groups based on *organization size*. The results indicate significant differences for awareness of a single threat. Post-hoc tests did not reveal any organization sizes that would be clearly distinguishable from others.

**Table 9:** Differences in awareness of threats across organization size groups.

	1: Micro	2: Small	3: Medium	4: Large	*H*	*p*	*p*_1−2_	*p*_1−3_	*p*_2−3_	*p*_1−4_	*p*_2−4_	*p*_3−4_
Loss of access to data (e.g., ransomware, locking of devices)												
Not aware	27(20.3%)	20(22.7%)	6(12.2%)	2(15.4%)	2.503	0.4748						
Aware	106(79.7%)	67(76.1%)	43(87.8%)	11(84.6%)								
Information system intrusion (e.g., hacking)												
Not aware	22(16.5%)	12(13.6%)	6(12.2%)	1(7.7%)	1.184	0.7569						
Aware	111(83.5%)	76(86.4%)	43(87.8%)	12(92.3%)								
Theft of business-critical data (industrial espionage)												
Not aware	42(31.6%)	26(29.5%)	8(16.3%)	1(7.7%)	6.931	0.0742						
Aware	91(68.4%)	62(70.5%)	41(83.7%)	12(92.3%)								
Distributed denial of service (DDoS) attacks												
Not aware	55(41.4%)	26(29.5%)	13(26.5%)	5(38.5%)	4.948	0.1757						
Aware	78(58.6%)	61(69.3%)	36(73.5%)	8(61.5%)								
Takeover of devices (e.g., botnets)												
Not aware	56(42.1%)	17(19.3%)	12(24.5%)	6(46.2%)	14.460**	0.0023	0.0034	0.1472	1.0000	1.0000	0.3513	0.8311
Aware	77(57.9%)	69(78.4%)	37(75.5%)	7(53.8%)								
Malware infection (e.g., viruses, worms, Trojans, spyware)												
Not aware	19(14.3%)	13(14.8%)	6(12.2%)	(0.0%)	2.284	0.5156						
Aware	114(85.7%)	74(84.1%)	42(85.7%)	13(100.0%)								
Phishing												
Not aware	39(29.3%)	19(21.6%)	10(20.4%)	1(7.7%)	4.501	0.2122						
Aware	94(70.7%)	69(78.4%)	39(79.6%)	12(92.3%)								
Online fraud (e.g., CEO fraud, business email compromise)												
Not aware	32(24.1%)	15(17.0%)	9(18.4%)	3(23.1%)	1.818	0.6110						
Aware	101(75.9%)	73(83.0%)	40(81.6%)	10(76.9%)								
Internal threats (e.g., deliberate data deletion, unauthorized data access, unauthorized personal devices)												
Not aware	33(24.8%)	14(15.9%)	5(10.2%)	2(15.4%)	6.515	0.0891						
Aware	96(72.2%)	73(83.0%)	44(89.8%)	11(84.6%)								

We conducted Kruskal-Wallis tests to determine whether there are differences in awareness of threats across groups. For threats with significant differences across groups, we conducted post-hoc Dunn’s tests with Bonferroni correction to determine which groups significantly differ from each other.

Notes:

*p < 0.05,

**p < 0.01,

***p < 0.001.

Table 10 presents the differences in respondents’ *awareness of solutions* for groups based on *organization size*. The results indicate significant differences for awareness of four solutions. Again, no organization size was clearly distinguishable from others for any of these solutions.

**Table 10:** Differences in awareness of solutions across organization size groups.

	1: Micro	2: Small	3: Medium	4: Large	*H*	*p*	*p*_1−2_	*p*_1−3_	*p*_2−3_	*p*_1−4_	*p*_2−4_	*p*_3−4_
Remote data deletion on lost or stolen devices												
Not aware	43(32.3%)	22(25.0%)	2(4.1%)	3(23.1%)	15.127**	0.0017	1.0000	0.0006	0.0406	1.0000	1.0000	0.9794
Aware	89(66.9%)	65(73.9%)	46(93.9%)	10(76.9%)								
Advanced antimalware solutions (with EDR/XDR capabilities)												
Not aware	57(42.9%)	29(33.0%)	8(16.3%)	7(53.8%)	12.977**	0.0047	0.7582	0.0057	0.3251	1.0000	0.9113	0.0811
Aware	74(55.6%)	58(65.9%)	40(81.6%)	6(46.2%)								
Secure connection (e.g., VPN)												
Not aware	26(19.5%)	19(21.6%)	6(12.2%)	(0.0%)	4.916	0.1780						
Aware	107(80.5%)	69(78.4%)	43(87.8%)	13(100.0%)								
Cloud synchronization of data												
Not aware	27(20.3%)	14(15.9%)	7(14.3%)	1(7.7%)	2.171	0.5378						
Aware	105(78.9%)	74(84.1%)	42(85.7%)	12(92.3%)								
Data backup												
Not aware	19(14.3%)	18(20.5%)	11(22.4%)	(0.0%)	5.193	0.1582						
Aware	113(85.0%)	70(79.5%)	37(75.5%)	13(100.0%)								
Centralized device management, including mobile device management (MDM)												
Not aware	49(36.8%)	15(17.0%)	7(14.3%)	4(30.8%)	15.219**	0.0016	0.0070	0.0124	1.0000	1.0000	1.0000	1.0000
Aware	83(62.4%)	72(81.8%)	42(85.7%)	9(69.2%)								
Advanced firewalls (with IPS/IDS capabilities)												
Not aware	34(25.6%)	15(17.0%)	7(14.3%)	3(23.1%)	3.992	0.2624						
Aware	98(73.7%)	73(83.0%)	42(85.7%)	10(76.9%)								
Training on secure use of devices												
Not aware	33(24.8%)	20(22.7%)	7(14.3%)	(0.0%)	5.963	0.1134						
Aware	99(74.4%)	68(77.3%)	41(83.7%)	13(100.0%)								
Multi-factor authentication (e.g., 2FA)												
Not aware	43(32.3%)	18(20.5%)	12(24.5%)	2(15.4%)	5.048	0.1683						
Aware	89(66.9%)	70(79.5%)	37(75.5%)	11(84.6%)								
Security operation center (SOC) 24/7												
Not aware	62(46.6%)	28(31.8%)	11(22.4%)	7(53.8%)	11.349**	0.0100	0.1903	0.0232	1.0000	1.0000	0.8095	0.2541
Aware	71(53.4%)	59(67.0%)	37(75.5%)	6(46.2%)								
Centralized management of software updates												
Not aware	44(33.1%)	20(22.7%)	8(16.3%)	2(15.4%)	7.143	0.0675						
Aware	88(66.2%)	67(76.1%)	41(83.7%)	11(84.6%)								
Organizational critical infrastructure access control												
Not aware	45(33.8%)	18(20.5%)	11(22.4%)	5(38.5%)	5.646	0.1302						
Aware	88(66.2%)	68(77.3%)	37(75.5%)	8(61.5%)								

We conducted Kruskal-Wallis tests to determine whether there are differences in awareness of solutions across groups. For solutions with significant differences across groups, we conducted post-hoc Dunn’s tests with Bonferroni correction to determine which groups significantly differ from each other.

Notes:

*p < 0.05,

**p < 0.01,

***p < 0.001.

Based on the above, we can conclude that organization size is not associated with awareness of neither threats nor solutions.

### 3.3 RQ2b: Are there differences in cybersecurity awareness of decision-makers across groups based on their personal characteristics?

#### 3.3.1 Gender

Table 11 shows the differences in respondents’ *awareness of threats* for groups based on their *gender*. The results indicate significant differences in awareness of five threats. Male respondents were significantly more aware of loss of access to data, industrial espionage, DDoS attacks, botnets and phishing than female respondents.

**Table 11:** Differences in awareness of threats across genders.

	Female	Male	*W*	*p*
Loss of access to data (e.g., ransomware, locking of devices)				
Not aware	25(28.7%)	30(15.4%)	7319.0**	0.0097
Aware	62(71.3%)	164(84.1%)		
Information system intrusion (e.g., hacking)				
Not aware	10(11.5%)	31(15.9%)	8856.0	0.3341
Aware	77(88.5%)	164(84.1%)		
Theft of business-critical data (industrial espionage)				
Not aware	31(35.6%)	46(23.6%)	7461.0*	0.0365
Aware	56(64.4%)	149(76.4%)		
Distributed denial of service (DDoS) attacks				
Not aware	43(49.4%)	56(28.7%)	6704.0***	0.0009
Aware	44(50.6%)	138(70.8%)		
Takeover of devices (e.g., botnets)				
Not aware	41(47.1%)	50(25.6%)	6614.0***	0.0005
Aware	46(52.9%)	143(73.3%)		
Malware infection (e.g., viruses, worms, Trojans, spyware)				
Not aware	16(18.4%)	22(11.3%)	7808.5	0.1149
Aware	71(81.6%)	171(87.7%)		
Phishing				
Not aware	32(36.8%)	37(19.0%)	6972.0**	0.0013
Aware	55(63.2%)	158(81.0%)		
Online fraud (e.g., CEO fraud, business email compromise)				
Not aware	19(21.8%)	40(20.5%)	8370.0	0.8016
Aware	68(78.2%)	155(79.5%)		
Internal threats (e.g., deliberate data deletion, unauthorized data access, unauthorized personal devices)				
Not aware	18(20.7%)	36(18.5%)	8042.0	0.6871
Aware	68(78.2%)	155(79.5%)		

We conducted Wilcoxon tests to determine whether there are differences in awareness of threats across groups.

Notes:

*p < 0.05,

**p < 0.01,

***p < 0.001.

Table 12 shows the differences in respondents’ *awareness of solutions* for groups based on their *gender*. The results indicate significant differences for awareness of six solutions. Male respondents were significantly more aware of advanced antimalware solutions with EDR/XDR capabilities, centralized device management, training, multi-factor authentication, centralized management of software updates and critical infrastructure access control than female respondents.

**Table 12:** Differences in awareness of solutions across genders.

	Female	Male	*W*	*p*
Remote data deletion on lost or stolen devices				
Not aware	26(29.9%)	44(22.6%)	7682.0	0.1871
Aware	60(69.0%)	149(76.4%)		
Advanced antimalware solutions (with EDR/XDR capabilities)				
Not aware	41(47.1%)	60(30.8%)	6900.0**	0.0086
Aware	45(51.7%)	132(67.7%)		
Secure connection (e.g., VPN)				
Not aware	18(20.7%)	33(16.9%)	8163.0	0.4494
Aware	69(79.3%)	162(83.1%)		
Cloud synchronization of data				
Not aware	16(18.4%)	33(16.9%)	8322.5	0.7793
Aware	71(81.6%)	161(82.6%)		
Data backup				
Not aware	16(18.4%)	32(16.4%)	8166.0	0.6671
Aware	70(80.5%)	162(83.1%)		
Centralized device management, including mobile device management (MDM)				
Not aware	32(36.8%)	43(22.1%)	7087.0**	0.0089
Aware	54(62.1%)	151(77.4%)		
Advanced firewalls (with IPS/IDS capabilities)				
Not aware	21(24.1%)	38(19.5%)	8055.0	0.3880
Aware	66(75.9%)	156(80.0%)		
Training on secure use of devices				
Not aware	27(31.0%)	33(16.9%)	7225.5**	0.0087
Aware	60(69.0%)	160(82.1%)		
Multi-factor authentication (e.g., 2FA)				
Not aware	33(37.9%)	42(21.5%)	7065.0**	0.0044
Aware	54(62.1%)	152(77.9%)		
Security operation center (SOC) 24/7				
Not aware	39(44.8%)	69(35.4%)	7633.5	0.1498
Aware	48(55.2%)	124(63.6%)		
Centralized management of software updates				
Not aware	34(39.1%)	40(20.5%)	6764.0***	0.0010
Aware	52(59.8%)	154(79.0%)		
Organizational critical infrastructure access control				
Not aware	34(39.1%)	45(23.1%)	6953.0**	0.0056
Aware	52(59.8%)	148(75.9%)		

We conducted Wilcoxon tests to determine whether there are differences in awareness of threats across groups.

Notes:

*p < 0.05,

**p < 0.01,

***p < 0.001.

These results suggest that male decision-makers are more aware of certain threats and solutions than their female counterparts. To be more specific, male decision-makers were more aware of five out of nine threats (55.6%), and six out of 12 solutions (50.0%).

#### 3.3.2 Formal education

Table 13 shows the differences in respondents’ *awareness of threats* for groups based on *formal education*. The results do not suggest any significant differences in awareness of threats among these groups.

**Table 13:** Differences in awareness of threats across formal education groups.

	Finished high school	Bachelor’s degree	Master’s degree	PhD degree	*H*	*p*
Loss of access to data (e.g., ransomware, locking of devices)						
Not aware	11(20.4%)	15(16.7%)	22(20.8%)	7(21.2%)	0.687	0.8764
Aware	43(79.6%)	75(83.3%)	83(78.3%)	26(78.8%)		
Information system intrusion (e.g., hacking)						
Not aware	6(11.1%)	9(10.0%)	20(18.9%)	6(18.2%)	3.951	0.2668
Aware	48(88.9%)	81(90.0%)	86(81.1%)	27(81.8%)		
Theft of business-critical data (industrial espionage)						
Not aware	15(27.8%)	24(26.7%)	24(22.6%)	14(42.4%)	4.978	0.1734
Aware	39(72.2%)	66(73.3%)	82(77.4%)	19(57.6%)		
Distributed denial of service (DDoS) attacks						
Not aware	21(38.9%)	33(36.7%)	36(34.0%)	9(27.3%)	1.350	0.7172
Aware	33(61.1%)	57(63.3%)	69(65.1%)	24(72.7%)		
Takeover of devices (e.g., botnets)						
Not aware	19(35.2%)	29(32.2%)	32(30.2%)	11(33.3%)	0.382	0.9440
Aware	35(64.8%)	60(66.7%)	73(68.9%)	22(66.7%)		
Malware infection (e.g., viruses, worms, Trojans, spyware)						
Not aware	10(18.5%)	10(11.1%)	12(11.3%)	6(18.2%)	2.548	0.4667
Aware	44(81.5%)	79(87.8%)	93(87.7%)	27(81.8%)		
Phishing						
Not aware	19(35.2%)	18(20.0%)	22(20.8%)	10(30.3%)	5.719	0.1261
Aware	35(64.8%)	72(80.0%)	84(79.2%)	23(69.7%)		
Online fraud (e.g., CEO fraud, business email compromise)						
Not aware	15(27.8%)	17(18.9%)	22(20.8%)	5(15.2%)	2.422	0.4896
Aware	39(72.2%)	73(81.1%)	84(79.2%)	28(84.8%)		
Internal threats (e.g., deliberate data deletion, unauthorized data access, unauthorized personal devices)						
Not aware	13(24.1%)	14(15.6%)	21(19.8%)	6(18.2%)	1.807	0.6135
Aware	40(74.1%)	76(84.4%)	82(77.4%)	26(78.8%)		

We conducted Kruskal-Wallis tests to determine whether there are differences in awareness of threats across groups.

Table 14 shows the differences in respondents’ *awareness of solutions* for groups based on *formal education*. These results also do not suggest any significant differences in awareness of solutions among these groups.

**Table 14:** Differences in awareness of solutions across formal education groups.

	Finished high school	Bachelor’s degree	Master’s degree	PhD degree	*H*	*p*
Remote data deletion on lost or stolen devices						
Not aware	17(31.5%)	21(23.3%)	24(22.6%)	8(24.2%)	1.532	0.6749
Aware	37(68.5%)	67(74.4%)	81(76.4%)	25(75.8%)		
Advanced antimalware solutions (with EDR/XDR capabilities)						
Not aware	26(48.1%)	32(35.6%)	33(31.1%)	10(30.3%)	5.307	0.1507
Aware	27(50.0%)	56(62.2%)	72(67.9%)	23(69.7%)		
Secure connection (e.g., VPN)						
Not aware	11(20.4%)	17(18.9%)	18(17.0%)	5(15.2%)	0.507	0.9173
Aware	43(79.6%)	73(81.1%)	88(83.0%)	28(84.8%)		
Cloud synchronization of data						
Not aware	8(14.8%)	17(18.9%)	18(17.0%)	6(18.2%)	0.456	0.9284
Aware	46(85.2%)	72(80.0%)	88(83.0%)	27(81.8%)		
Data backup						
Not aware	6(11.1%)	11(12.2%)	22(20.8%)	9(27.3%)	6.071	0.1082
Aware	48(88.9%)	77(85.6%)	84(79.2%)	24(72.7%)		
Centralized device management, including mobile device management (MDM)						
Not aware	20(37.0%)	21(23.3%)	26(24.5%)	8(24.2%)	4.080	0.2529
Aware	33(61.1%)	68(75.6%)	80(75.5%)	25(75.8%)		
Advanced firewalls (with IPS/IDS capabilities)						
Not aware	11(20.4%)	17(18.9%)	23(21.7%)	8(24.2%)	0.445	0.9308
Aware	43(79.6%)	72(80.0%)	83(78.3%)	25(75.8%)		
Training on secure use of devices						
Not aware	15(27.8%)	15(16.7%)	20(18.9%)	10(30.3%)	4.248	0.2359
Aware	39(72.2%)	73(81.1%)	86(81.1%)	23(69.7%)		
Multi-factor authentication (e.g., 2FA)						
Not aware	15(27.8%)	28(31.1%)	24(22.6%)	8(24.2%)	2.053	0.5615
Aware	39(72.2%)	61(67.8%)	82(77.4%)	25(75.8%)		
Security operation center (SOC) 24/7						
Not aware	25(46.3%)	32(35.6%)	43(40.6%)	8(24.2%)	4.715	0.1939
Aware	29(53.7%)	57(63.3%)	62(58.5%)	25(75.8%)		
Centralized management of software updates						
Not aware	17(31.5%)	17(18.9%)	32(30.2%)	8(24.2%)	4.172	0.2435
Aware	36(66.7%)	72(80.0%)	74(69.8%)	25(75.8%)		
Organizational critical infrastructure access control						
Not aware	18(33.3%)	21(23.3%)	32(30.2%)	8(24.2%)	2.316	0.5095
Aware	35(64.8%)	68(75.6%)	73(68.9%)	25(75.8%)		

We conducted Kruskal-Wallis tests to determine whether there are differences in awareness of solutions across groups.

Based on the above, we can conclude that formal education is not associated with cybersecurity awareness of decision-makers.

#### 3.3.3 Age

Table 15 shows the differences in respondents’ *age* for groups based on *awareness of threats*. The results reveal significant differences in average age for awareness of three threats (i.e., industrial espionage, malware infection and phishing). Respondents who were aware of these threats were significantly older than those who were not.

**Table 15:** Differences in age across groups based on awareness of threats.

	Not aware	Aware	*t*	*p*
Loss of access to data (e.g., ransomware, locking of devices)	34.42	37.24	−1.557	0.1205
Information system intrusion (e.g., hacking)	35.66	36.83	−0.576	0.5650
Theft of business-critical data (industrial espionage)	33.55	37.83	−2.689**	0.0076
Distributed denial of service (DDoS) attacks	37.31	36.35	0.640	0.5224
Takeover of devices (e.g., botnets)	35.54	37.23	−1.090	0.2766
Malware infection (e.g., viruses, worms, Trojans, spyware)	32.37	37.26	−2.354*	0.0193
Phishing	33.81	37.59	−2.276*	0.0236
Online fraud (e.g., CEO fraud, business email compromise)	37.24	36.51	0.411	0.6817
Internal threats (e.g., deliberate data deletion, unauthorized data access, unauthorized personal devices)	36.33	36.76	−0.233	0.8156

We conducted independent samples *t* tests to determine whether there are differences in age across groups.

Notes:

*p < 0.05,

**p < 0.01,

***p < 0.001.

Table 16 shows the differences in respondents’ *age* for groups based on *awareness of solutions*. The results reveal significant differences in average age for awareness of two solutions (i.e., cloud synchronization of data and data backup). Respondents who were aware of these solutions were significantly older than those who were not.

**Table 16:** Differences in age across groups based on awareness of solutions.

	Not aware	Aware	*t*	*p*
Remote data deletion on lost or stolen devices	36.17	37.01	−0.502	0.6157
Advanced antimalware solutions (with EDR/XDR capabilities)	37.50	36.27	0.819	0.4133
Secure connection (e.g., VPN)	33.92	37.27	−1.800	0.0729
Cloud synchronization of data	32.73	37.56	−2.570*	0.0107
Data backup	31.96	37.47	−2.940**	0.0036
Centralized device management, including mobile device management (MDM)	37.68	36.41	0.777	0.4377
Advanced firewalls (with IPS/IDS capabilities)	35.78	36.96	−0.670	0.5033
Training on secure use of devices	34.42	37.23	−1.616	0.1073
Multi-factor authentication (e.g., 2FA)	36.35	36.85	−0.302	0.7628
Security operation center (SOC) 24/7	38.24	35.58	1.813	0.0710
Centralized management of software updates	36.07	36.84	−0.473	0.6369
Organizational critical infrastructure access control	36.75	36.59	0.098	0.9219

We conducted independent samples *t* tests to determine whether there are differences in age across groups.

Notes:

*p < 0.05,

**p < 0.01,

***p < 0.001.

These results indicate that decision-makers who are aware of certain threats and solutions are older than decision-makers who are not. However, this is true only for a minority of threats (33.3%) and solutions (16.7%) included in our study.

#### 3.3.4 Experience with information security

Table 17 presents the differences in respondents’ *experience with information security* for groups based on *awareness of threats*. The results show significant differences in average experience with information security for awareness of four threats (i.e., loss of access to data, DDoS attacks, malware infection and phishing). Respondents who were aware of these threats had significantly more experience with information security than those who were not.

**Table 17:** Differences in experience with information security across groups based on awareness of threats.

	Not aware	Aware	*t*	*p*
Loss of access to data (e.g., ransomware, locking of devices)	7.52	10.50	−2.665**	0.0092
Information system intrusion (e.g., hacking)	8.74	10.13	−0.991	0.3225
Theft of business-critical data (industrial espionage)	8.37	10.51	−1.968	0.0501
Distributed denial of service (DDoS) attacks	8.57	10.65	−2.026*	0.0437
Takeover of devices (e.g., botnets)	8.70	10.54	−1.752	0.0809
Malware infection (e.g., viruses, worms, Trojans, spyware)	7.03	10.25	−2.554*	0.0137
Phishing	8.14	10.50	−2.083*	0.0382
Online fraud (e.g., CEO fraud, business email compromise)	8.07	10.40	−1.940	0.0534
Internal threats (e.g., deliberate data deletion, unauthorized data access, unauthorized personal devices)	8.48	10.26	−1.437	0.1519

We conducted independent samples *t* tests to determine whether there are differences in experience with information security across groups.

Notes:

*p < 0.05,

**p < 0.01,

***p < 0.001.

Table 18 presents the differences in respondents’ *experience with information security* for groups based on *awareness of solutions*. The results show significant differences in average experience with information security for awareness of seven solutions (i.e., remote data deletion, advanced antimalware solutions with EDR/XDR capabilities, secure connection, cloud synchronization of data, centralized device management, advanced firewalls and training). Respondents who were aware of these solutions had significantly more experience with information security than those who were not.

**Table 18:** Differences in experience with information security across groups based on awareness of solutions.

	Not aware	Aware	*t*	*p*
Remote data deletion on lost or stolen devices	8.21	10.51	−2.045*	0.0419
Advanced antimalware solutions (with EDR/XDR capabilities)	8.11	10.83	−2.919**	0.0039
Secure connection (e.g., VPN)	6.90	10.60	−3.318**	0.0014
Cloud synchronization of data	7.41	10.44	−2.332*	0.0204
Data backup	8.70	10.05	−1.060	0.2901
Centralized device management, including mobile device management (MDM)	7.63	10.75	−2.824**	0.0051
Advanced firewalls (with IPS/IDS capabilities)	7.60	10.51	−2.410*	0.0166
Training on secure use of devices	7.20	10.54	−3.205**	0.0018
Multi-factor authentication (e.g., 2FA)	9.22	10.19	−0.889	0.3750
Security operation center (SOC) 24/7	9.04	10.30	−1.286	0.1994
Centralized management of software updates	8.51	10.45	−1.713	0.0878
Organizational critical infrastructure access control	8.78	10.25	−1.381	0.1684

We conducted independent samples *t* tests to determine whether there are differences in experience with information security across groups.

Notes:

*p < 0.05,

**p < 0.01,

***p < 0.001.

These results indicate that decision-makers who are aware of approximately a half of threats (44.4%) and solutions (58.3%) included in our study have more experience with information security than decision-makers who are not.

#### 3.3.5 Experience with IT

Table 19 shows the differences in respondents’ *experience with IT* for groups based on *awareness of threats*. The results indicate significant differences in average experience with IT for awareness of six threats (i.e., loss of access to data, industrial espionage, DDoS attacks, botnets, malware infection and phishing). Respondents who were aware of these threats had significantly more experience with IT than those who were not.

**Table 19:** Differences in experience with IT across groups based on awareness of threats.

	Not aware	Aware	*t*	*p*
Loss of access to data (e.g., ransomware, locking of devices)	7.36	11.17	−2.959**	0.0034
Information system intrusion (e.g., hacking)	8.95	10.67	−1.165	0.2451
Theft of business-critical data (industrial espionage)	7.84	11.38	−3.374***	0.0009
Distributed denial of service (DDoS) attacks	8.89	11.25	−2.189*	0.0294
Takeover of devices (e.g., botnets)	8.51	11.38	−2.618**	0.0093
Malware infection (e.g., viruses, worms, Trojans, spyware)	6.86	10.85	−3.076**	0.0034
Phishing	8.08	11.16	−2.585*	0.0102
Online fraud (e.g., CEO fraud, business email compromise)	8.93	10.81	−1.486	0.1385
Internal threats (e.g., deliberate data deletion, unauthorized data access, unauthorized personal devices)	8.38	10.83	−1.863	0.0636

We conducted independent samples *t* tests to determine whether there are differences in experience with IT across groups.

Notes:

*p < 0.05,

**p < 0.01,

***p < 0.001.

Table 20 presents the differences in respondents’ *experience with IT* for groups based on *awareness of solutions*. The results show significant differences in average experience with IT for awareness of eight solutions (i.e., remote data deletion, advanced antimalware solutions with EDR/XDR capabilities, secure connection, cloud synchronization of data, data backup, centralized device management, advanced firewalls and training). Respondents who were aware of these solutions had significantly more experience with IT than those who were not.

**Table 20:** Differences in experience with IT across groups based on awareness of solutions.

	Not aware	Aware	*t*	*p*
Remote data deletion on lost or stolen devices	8.44	11.10	−2.235*	0.0262
Advanced antimalware solutions (with EDR/XDR capabilities)	8.67	11.30	−2.501*	0.0130
Secure connection (e.g., VPN)	7.08	11.16	−3.106**	0.0021
Cloud synchronization of data	7.39	11.03	−2.664**	0.0082
Data backup	7.83	10.81	−2.219*	0.0273
Centralized device management, including mobile device management (MDM)	7.70	11.39	−3.172**	0.0017
Advanced firewalls (with IPS/IDS capabilities)	7.71	11.11	−2.686**	0.0077
Training on secure use of devices	6.85	11.26	−4.192***	0.0000
Multi-factor authentication (e.g., 2FA)	9.41	10.80	−1.200	0.2312
Security operation center (SOC) 24/7	9.60	10.78	−1.138	0.2563
Centralized management of software updates	8.93	10.94	−1.698	0.0907
Organizational critical infrastructure access control	9.18	10.79	−1.421	0.1564

We conducted independent samples *t* tests to determine whether there are differences in experience with IT across groups.

Notes:

*p < 0.05,

**p < 0.01,

***p < 0.001.

These results indicate that decision-makers who are aware of two thirds of threats (66.7%) and solutions (66.7%) included in our study have more experience with IT than decision-makers who are not.

## 4 Discussion

In this section, we first provide theoretical and practical implications of this study. Next, we discuss its limitations and put forward directions for future research.

### 4.1 Theoretical implications

This study makes a number of theoretical contributions to the literature. First, awareness of well-known threats and solutions seems to be quite low for cyber and information security decision-makers. The results of this study indicate that about a third of decision-makers are not aware of DDoS attacks and botnets, and about a quarter are not aware of industrial espionage and phishing. This is especially surprising since these threats are among the currently most prevalent ones [38, 40]. Awareness of solutions does not appear to be much better. A quarter or more decision-makers are not aware of seven out of 12 solutions included in our study. More than a third of decision-makers were unaware of two of these solutions, namely SOC and advanced antimalware solutions with EDR/XDR capabilities, and a quarter or more decision-makers were unaware of further five solutions (i.e., organizational critical infrastructure access control, centralized device management, including mobile device management (MDM), multi-factor authentication (e.g., 2FA), centralized management of software updates, remote data deletion on lost or stolen devices). Even though cyber and information security decision-makers are the primary enablers of cybersecurity in organizations, the results of our study suggest they are insufficiently aware of both threats and solutions. This lack of decision-makers’ cybersecurity awareness may significantly impact their ability to make adequate decisions regarding cybersecurity, which is a challenging task to start with [9]. Additionally, this may hinder their ability to lead their organizations towards cyber-resilient culture [11, 31]. These findings are in line with most published literature (e.g., [33, 34]). This study makes a contribution to the literature on awareness of decision-makers by breaking down cybersecurity awareness into various kinds of threats and solutions. This break-down view shows that cybersecurity awareness may not be a monolithic construct thus future studies may incorporate its different dimensions in their research designs.

Second, this study suggests that there are differences in cybersecurity awareness of decision-makers across groups based on adoption of antimalware solutions in their organizations. The results of our study indicate that awareness of certain but not all threats and solutions is positively associated with either adoption of antimalware solution types or adoption of SOC realizations. These findings contribute to the literature on awareness of decision-makers in organizations adopting different types of advanced antimalware solutions. We need to note that we did not search for causal relationships in our study. Therefore, it remains unclear whether higher awareness of decision-makers in organizations adopting advanced antimalware solutions with EDR/XDR capabilities or organizations adopting either an internal or external SOC is the consequence of this adoption or vice versa. Future studies may thus focus on investigating this causal relationship.

Third, the results of our study indicate that there are differences in cybersecurity awareness of decision-makers across their organizational role types. Non-IT/IS executive decision-makers had the least awareness which may be the consequence of their background, and appears to be in line with existing literature [48]. Next, non-executive decision-makers seem to be more aware of certain solutions than other decision-makers which may be a consequence of their more operational involvement in ensuring cybersecurity. This adds to the literature as one of the first studies to investigate the importance of organizational role types. The results of our study however do not support the role of organizational size as suggested by published literature [29, 34]. The reason for this divergence could be found in our target population which included decision-makers at both executive and non-executive levels, and with varying backgrounds (i.e., IT/IS and non-IT/IS). Future studies may focus on each type of organizational role type individually.

Fourth, our study identifies several personal characteristics of decision-makers associated with their cybersecurity awareness. Gender seems to be the most important demographic associated with cybersecurity awareness of decision-makers. Similarly to the published literature [48], male decision-makers were more aware than their female counterparts. Age was also associated with cybersecurity awareness although for a much lower share of threats and solutions. Contrary to the published literature [34], formal education was not a significant factor in cybersecurity awareness of decision-makers. Experience with IT was the most seminal personal characteristic associated with cybersecurity awareness of decision-makers, surpassing the share of significant threats and solutions compared to experience with information security. Although this is somewhat surprising, the importance of IT has been emphasized in the literature before [30]. These findings contribute to the literature on the relation between personal characteristics of decision-makers and their cybersecurity awareness. Future works may consider incorporating these characteristics in their research models.

### 4.2 Practical implications

This paper provides some practical implications for increasing the ability to achieve higher levels of cyber-resilience in organizations. Since cyber and information security decision-makers are the main drivers and enablers of the cybersecurity mindset in organizations, understanding and improving their cybersecurity awareness has the potential to further improve the overall cybersecurity of organizations. First, this study provides insights into which threats and solutions are less known among cyber and information security decision-makers. Therefore, it provides straight-forward guidance on which threats and solutions need to be better promoted among cyber and information security decision-makers.

Second, the results of our study identify which decision-makers are more likely to be less aware of threats and solutions—i.e., especially decision-makers in organizations not adopting a malware solution, decision-makers in organizations not adopting a SOC, non-IT/IS executive decision-makers, female and younger decision-makers, and decision-makers with less experience with IT or information security. These characteristics can help to target the most needy decision-makers with cybersecurity awareness interventions. Detailed insights from this study may additionally help to adapt such interventions to the needs of a specific subgroup of decision-makers.

### 4.3 Limitations and future research

This study has some limitations that the readers should note. First, the study was conducted in a single cultural context. Since cultural contexts may be an important factor when studying cybersecurity awareness, the findings of this study may not be generalized to other cultural contexts. Future studies comparing our findings to or investigating the influence of different cultural contexts would help to further generalize the findings of our study. Second, the questionnaire included single items for measured constructs, such as dimensions of threats and solutions. This significantly affects the ability to check reliability and validity of the measurement instrument. Although we addressed this issue by merging scores for both awareness constructs, future studies may be needed to further confirm our findings. Third, we are unsure whether there were cases of more than one respondent from a single organization since we did not ask respondents to name organizations in which they were employed. If there were several such cases, it may partially influence the results related to organizational factors, such as *adopted antimalware solution type*, *adopted SOC realization*, and *organization size*. Even though the possibility of this are relatively low, future studies would be beneficial for confirming the findings of our study. Fourth, this study focuses on a single countermeasure (i.e., antimalware solutions). Future studies may explore differences in cybersecurity awareness of decision-makers based on adoption of other countermeasures (e.g., [45]). Fifth, we opted for a cross-sectional survey research design to reach more respondents than with alternatives. This enabled us to get a broader overview of the studied topic albeit at the cost of the depth of insights for each respondent. Alternative research designs employing other research methods, such as interviews or focus groups, may provide deeper insights for each respondent.

## Supporting information

S1 Dataset Survey data. (CSV)

S1 File Legend for survey data. (ODS)

S2 File R script for data analysis. (R)
